# Editorial: Enabling Biomaterials for New Biomedical Technologies and Clinical Therapies

**DOI:** 10.3389/fbioe.2020.00559

**Published:** 2020-06-05

**Authors:** Hasan Uludag, Abhay Pandit, Liisa Kuhn

**Affiliations:** ^1^Department of Chemical and Materials Engineering, University of Alberta, Edmonton, AB, Canada; ^2^Faculty of Pharmacy & Pharmaceutical Sciences, Edmonton, AB, Canada; ^3^Biomedical Engineering, Faculty of Medicine and Dentistry, Edmonton, AB, Canada; ^4^CÚRAM, SFI Research Centre for Medical Devices, National University of Ireland Galway, Galway, Ireland; ^5^Department of Biomedical Engineering, University of Connecticut Health Center, Farmington, CT, United States

**Keywords:** biomaterials, biocompatibility, biomaterial-host interface, regeneration, metallic implants, safety, tissue engineering, therapeutic

Biomaterials have been an integral part of exciting developments in medicine. They singlehandedly enabled new therapeutic modalities and gave new life to diagnostic procedures as well as supporting innovative developments in medicine. In anticipation of the future developments in the field, we compiled this Research Topic to provide complementary perspectives from biomaterials researchers into emerging concepts in biomaterials science and engineering. The Research Topic is intended to summarize the latest biomaterials advances in emerging biomedical technologies and effective interventions promising for the clinical setting. It has the ambitious goal of broadly tackling key challenges facing biomaterials researchers. The well-established working hypothesis, that is *material chemistry influences genetic flow of information in a cell, which in turn alters cellular behavior in contact with biomaterial, leading to organ level changes*, are central in this Research Topic. Individual manuscripts in this collection represents a combination of in-depth reviews, technical studies, and perspectives from leading experts in their fields, summarizing the-state-of-the-art in critical aspects of biomaterials. The Research Topic has a relatively broad theme to allow information exchange from different aspects of the field. The experts were encouraged to highlight their recent work and identify key developments with transformative potential.

Given the central role of “biocompatibility” in the field, the hypothesis article by Williams explored the “*specifications for innovative, enabling biomaterials based on the principles of biocompatibility mechanisms*.” It has been recognized that “efficacy” and “safety” are two indispensable aspects of biomaterials (Uludag, 2014) and this article concentrates on the under-studied issue of “biocompatibility” that determines the acute and chronic interactions with the host. The article takes a case-by-case approach and explores biocompatibility in leading applications of biomaterials; some applications are relatively simple (e.g., contrast agents) while others (e.g., tissue engineering) involve complicated set of tissue events for a successful outcome. As the author rightfully calls for a clearer understanding of the mechanisms of biocompatibility pathways within the context of applications, the importance of manipulating various pathways have been emphasized to enhance the chances of designing new and improved functional biomaterials.

Eltit et al. also take on the biocompatibility challenge at the interface of hip implants, focusing on metallic implants and the mechanisms of adverse local tissue reactions. Metallic biomaterials, being as artificial as they get for organic life-forms like us, most likely elicit unique reactions in the host tissue upon corrosion and release of by-products. Alternatively, non-specific effects involving general toxicity and inflammatory reactions as a result of local host cell death may elicit a response common with other non-metallic biomaterials. Careful analysis of unique vs. common aspects of the adverse local tissue reactions may lend itself to not only improved metallic implants but also to implants from biomaterials beyond the metallic kind. Considering the great interest in developing metallic micro/nano-scale constructs (with elevated surface areas) in diagnostic and therapeutic utilities, the importance of metallic biocompatibility will be even greater. Focusing on the leading metallic implant, titanium, Hanawa inspects the titanium-host interfaces from the perspective of controlling the interface reaction based on engineered features. With the ultimate goal of improved osseointegration, the review lays out different approaches to achieve most desirable porous surfaces with physical, chemical and biological features to encourage integrative events and discourage inhibitory actions. As in Eltit et al. and Hanawa calls for the need for better understanding the mechanism of biocompatibility and adverse events, despite extensive medical use of titanium over the years. Key issues remain to be better clarified that will be important for future applications of this important metallic biomaterial.

The critical issue of host reaction and in particular its control for success of immune-isolation devices has been also tacked by Hu and de Vos. The authors explore the choice of polymeric coatings and crosslinking agents that induce more stable and biocompatible isolation barriers, ultimately helping to attenuate the undesirable host response. Contributions of protein adsorption on surfaces intertwined with the immune stimulation from the devices were framed with engineered interfaces to control host reactions. A perspective on cellular approaches to mediate host response could provide clues for the design of more advanced materials and immune-isolation barriers that rely on biomimicry. Bu et al. also focused on biocompatibility issues in the context of aliphatic polyesters, a class of widely used synthetic polymers that includes poly(lactide/glycolide) family of polymers with long history of medical use. Key physicochemical features of aliphatic ester surfaces are succinctly presented including surface morphology, while considerations for engineering of surfaces from chemical and physical perspective are laid out. The information from this class of popular biomaterials should guide efforts when developing novel approaches for the biomedical use of such biomaterials.

Iron oxide is another metallic biomaterial that is finding unique applications in nanoparticulate form due its inherent magnetic properties (Dadfar et al., 2019). The science and technology of iron oxide magnetic nanoparticles are presented in a review article by Xu et al. in the context of bacterial detection and eradication. Different aspects of this specific application including bacterial enrichment, bioimaging and targeted delivery of anti-bacterial drugs and hyperthermia induction are articulated. A wide range of microbial targeting agents including antibodies, molecular entities acting as antimicrobial agents, peptides, bacteriophages, and aptamers are highlighted in order to create unique technologies in the context of controlling microbial growth.

The opposite end of the biomaterial spectrum is occupied by the naturally-derived collagen that has no physical, chemical and biological similarity to metallic biomaterials. In line with the mantra of “like attracts like,” Copes et al. explore the use of collagen, the ubiquitous component of the extracellular matrix, in vascular tissue-engineered grafts. The presence of inherent biological clues make collagen a no-brainer to rely on for modifying cellular activities *in situ*. The technology of collagen coatings as cell-substrates and as controlled release vehicles are explored. Industrial processing of collagen is complicated due to its heterogeneous composition (with a mix of contrasting functional groups) and excessive denaturation that may be encountered during sourcing to obtain pure formulations. Critical technological issues and limitations of collagen are carefully laid in this review, especially, when one attempts to create *de novo* vascular grafts *ex vivo*. In contrast to this philosophy, the original research article by Pohan et al. explored the synthetic polymer poly(vinyl alcohol) to create a tissue-engineered vascular graft; simple amine groups were grafted onto the luminal surface of the synthetic matrix using radio frequency glow discharge (RFGD) treatment, leading to a functional substrate for endothelial cells. Distinct differences from collagen coatings were noted in a baboon *ex vivo* shunt model, where the engineered grafts did not invoke platelet or fibrin activation in short-term studies.

Biomaterials capable to stimulating tissue repair is especially in demand for tissues with limited regenerative capacity, among which the neural tissues are most notable. Toward this goal Motamed et al. designed an RGD-bearing β-peptide hydrogel to aid the migration of neural stem cells originating from the subventricular zone within the brain tissue of an experimental model. Local delivery of Brain Derived Neurotrophic Factor (BDNF) by the hydrogels assisted with reducing the local inflammatory response, supported better host tissue-scaffold integration, and attracted astrocytes that aided with the differentiation of neuroblasts. The review article by Hosoyama et al. further explore studies focused on peptide-based approaches to create structural recognition motifs to enhance cellular attachment and induce cell signaling for functional outcomes. Current approaches to design and applications of short mimetic peptides for angiogenic, anti-inflammatory, and adhesive responses are articulated along with specific applications to better soft-tissue healing. It is evident that, starting with the simple cell-adhesive RGD technology (Huettner et al., 2018), peptide-mediated signaling is continuing to play more prominent roles in constructing functional tissues *ex vivo* and regenerative medicine *in vivo*.

Dental implantology is another area that will benefit from control of regenerative events for longer lasting interventions. Dental implants were probably the first implanted biomaterials stretching prior to recorded history, but there is still a need for novel approaches and functional biomaterials for longer lasting, more stable implants. The review article by Raddall et al. focuses on pulp-dentin interface and articulates the role of biomaterial scaffolds to support mesenchymal stem cell mediated regenerative endodontic therapy. Exploring scaffolds derived from host or exogenous sources (either synthetic or naturally derived), the article stipulates desirable features of biomaterials scaffolds and provides a glimpse of what is to come in scaffold design in endodontic repairs. The articulated ideas are likely to be extended to regenerative events at other interfaces formed by different types of tissues (Patel et al., 2018).

Widely different and imaginative fabrication technologies are taking the field by storm. Static and dynamic constructs (so called 3D and 4D printing, respectively) are emerging fabrication techniques and Tamay et al. are providing a glimpse of the possibilities based on polymeric “printing” biomaterials. Originally rooted in manufacturing discipline, printing technology is reviewed in this article with special emphasis on the use of biologically acceptable materials and incorporating bioactive agents, including viable cells, in the printed constructs. The future of printing is going to rely on novel biomaterials with specific properties and this review provides ample avenues in the pursuit of such functional biomaterials, interwoven with specific applications that will equip the reader to match the biomaterials to applications at hand. A related bottom-up approach for tissue engineering and regenerative medicine is to deploy nanoparticles to control cellular events that was not possible in the recent past. Fathi-Achachelouei et al. describes the feasibility of spatial and temporal control of regenerative events based on nanoparticulate delivery of multiple growth factors and control of scaffold properties, along with the possibilities of diagnostic/therapeutic imaging. The spectrum of biomaterials used for nanoparticle preparation are presented, the authors emphasizing the unique features of each class of materials. Information on nanoparticle-based printing further complements the review by Tamay et al. and lays the groundwork for cost-effective therapies on the long run.

It is convenient for practitioners, albeit presumptuous, to ignore individual differences among the hosts (i.e., patients) and develop “generic” therapies with “one-size-fits-all” philosophy. However, focus on individual patients is paramount to comprehend outcomes in specific cases, and improve performance among the population while providing new approaches and mechanistic insights for novel substitutes. This is nowhere more visible than cancer therapeutics where “heterogeneity” in the disease is inherent and adaptation to intervention is the norm. The review article by Bray et al. focuses on addressing patient specificity in engineering tumor models *ex vivo*. Integrating distinct fields of cellular/molecular biology, extracellular matrix biology and scaffold design, this review summarizes exciting developments in 3D fabrication of devices to investigate fundamental issued related to tumor therapy (such as drug response and metastasis). Heterogeneity is central to this pursuit, and biomaterial scaffold-centered approaches for tissue mimics with advanced analytical tools including computational modeling and simulations will make greater impact, perhaps even obviate the animal models, before therapeutic progression to patients.

The use of bioactive glasses especially doped with strontium (Sr) has been reviewed by Kargozar et al. for bone repair. While there is an ongoing debate on the exact mechanism of beneficial effect for Sr doping, better functional outcomes on bone repair is stimulating research with these biomaterials, so that the authors provide a summary of beneficial effects of Sr-doped bioactive glasses in bone repair models along with the current state of clinical use. The review summarizes the important facets of bioactive glasses, including their synthesis and atomistic details of their structural features. The technology for fabricating different bioactive glass constructs is presented along with possible mechanism(s) of action, ultimately leading to a comprehensive glimpse into the use of this unique type of biomaterials.

Continuing with the unique applications, Mobaraki et al. summarize the recent concepts and future prospects on the use of biomaterials for corneal tissue repair and regeneration. An interwoven array of pharmacological, cellular, and biomaterial based interventions is presented, which is bound to stimulate new cross-disciplinary approaches to corneal repair. New biomaterials relying on novel mechanisms of actions may emerge in this area based on mechanistic perspective provided in this review.

Calcium/phosphate nanoparticles are attractive for patient use since their breakdown products are simple ions that can be ready metabolized with no adverse effects. To visualize them, however, is challenging given the “interference” from the mineralized tissues. To overcome this problem, Kalidoss et al. describes calcium-deficient hydroxyapatite nanoparticles with silver, gadolinium, and iron substitutions to improve contrast in computed tomography. The nanoparticles, due to strong interfacial charges, were also beneficial to deliver therapeutic agents in a sustained manner, additionally enabling therapeutic effects beyond their possible diagnostic use.

Finally, the deployment of biomaterials for development of safe and effective non-viral gene delivery is reviewed by Uludag et al., which highlighted selected aspects of gene delivery efforts where the biomaterials are making an impact. Recent understandings of how cells utilize nucleic acids has led to a review of the types of nucleic acids that can be utilized for therapy. Given the excitement with recent T-cell based therapies, biomaterial-centered approaches (instead of viral approaches) to enable more effective and safer therapy are probed in this review. Authors' perspectives on designing intelligent nanoparticles, deploying mRNA as an alternative to plasmid DNA, long-acting (integrating) expression systems, and *in vitro*/*in vivo* expansion of engineered T-cells can be found in this review.

One can easily see from the summaries of the individual contributions that the compiled articles are broad in nature, emphasizing the science and technology of the explored topics. This was the intent, again, of this Research Theme where different aspects of biomaterials science and engineering have been painted with broad brush strokes. Cross-fertilization of new ideas are likely to emanate by compiling such complementary but not necessarily overlapping articles.

## Author Contributions

HU drafted the manuscript. LK and AP edited and proof-read the manuscript.

## Conflict of Interest

HU is the founder and share holder in RJH Biosciences intended to commercialize transfection reagents. The remaining authors declare that the research was conducted in the absence of any commercial or financial relationships that could be construed as a potential conflict of interest.
